# Fast and Accurate Semiautomatic Segmentation of Individual Teeth from Dental CT Images

**DOI:** 10.1155/2015/810796

**Published:** 2015-08-27

**Authors:** Ho Chul Kang, Chankyu Choi, Juneseuk Shin, Jeongjin Lee, Yeong-Gil Shin

**Affiliations:** ^1^Palette Soft Inc., 599 Kwanak-ro, Kwanak-gu, Seoul 151-742, Republic of Korea; ^2^Planet SK Planet Co., Ltd., Bundang-gu, 264 Pangyo-ro, Seongnam-si, Gyeonggi-do 463-400, Republic of Korea; ^3^Department of Systems Management Engineering, Sungkyunkwan University, 2066 Seobu-ro, Jangan-gu, Suwon-si, Gyeonggi-do 440-746, Republic of Korea; ^4^School of Computer Science & Engineering, Soongsil University, 369 Sangdo-ro, Dongjak-gu, Seoul 156-743, Republic of Korea; ^5^School of Computer Science and Engineering, Seoul National University, 599 Kwanak-ro, Kwanak-gu, Seoul 151-742, Republic of Korea

## Abstract

DIn this paper, we propose a fast and accurate semiautomatic method to effectively distinguish individual teeth from the sockets of teeth in dental CT images. Parameter values of thresholding and shapes of the teeth are propagated to the neighboring slice, based on the separated teeth from reference images. After the propagation of threshold values and shapes of the teeth, the histogram of the current slice was analyzed. The individual teeth are automatically separated and segmented by using seeded region growing. Then, the newly generated separation information is iteratively propagated to the neighboring slice. Our method was validated by ten sets of dental CT scans, and the results were compared with the manually segmented result and conventional methods. The average error of absolute value of volume measurement was 2.29 ± 0.56%, which was more accurate than conventional methods. Boosting up the speed with the multicore processors was shown to be 2.4 times faster than a single core processor. The proposed method identified the individual teeth accurately, demonstrating that it can give dentists substantial assistance during dental surgery.

## 1. Introduction

In the present, CT (Computed Tomography) and MRI (Magnetic Resonance Image) three-dimensional scans are developing with growing reliability, so they are expected to be used continuously in the various medical fields of the future. In particular, three-dimensional images are frequently used for braces and implant procedures in the field of dentistry [1–3]. For example, dentists are able to show the patients the plan and future image after completing the braces, before the patients are started on the braces. Moreover, three-dimensional images help both dentists and patients to understand the implant procedures intuitively. In this process, it is necessary to first detect the region of the maxilla or mandible [4, 5]. Then, the individual teeth are extracted from it independently, and the separation of individual teeth in the scans is required [6]. Although the separation of individual teeth is extremely important [7], it is very hard to tell the difference in the brightness between the actual teeth and the sockets of teeth in most of the images. Distinguishing individual teeth from others requires expertise as well as a long period of time [8]. There have been a number of researches on the automatic segmentation between teeth and sockets, but accuracy and stability of the segmentation were insufficient considering the segmentation speed [6, 7, 9].

**Figure 1:**
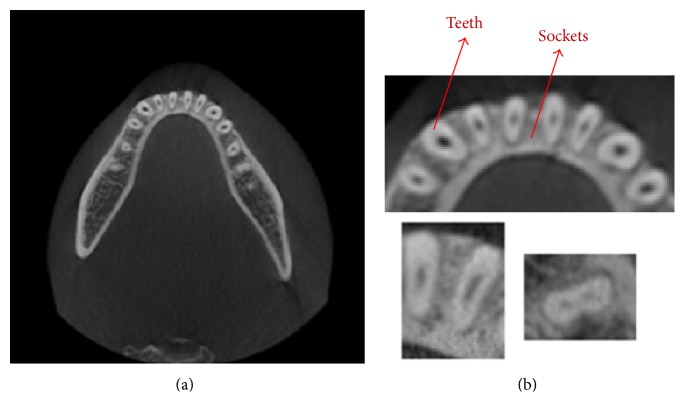
The axial view of the human mandible which is extracted with a threshold value: (a) is the original image of CT and (b) is the magnified view of the original image.

**Figure 2:**
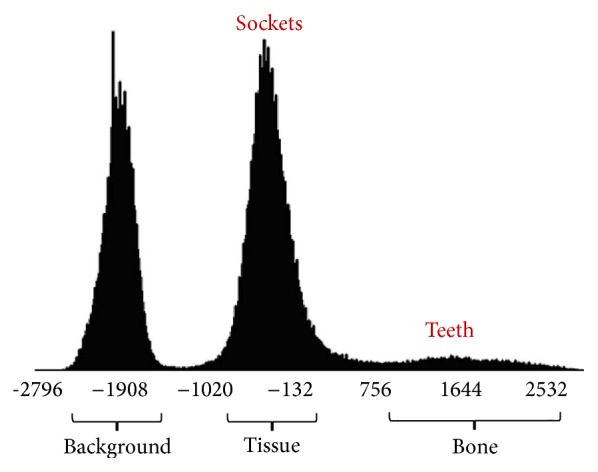
The histogram of CT scans of the mandible. It shows the dark background, tissues, and bones in the order from the darkest to the lightest.

Figure 1 shows the result of an axial view (2D slice image scanned along the *z*-axis) of the human mandible, which can be extracted with a threshold value of 1400 HU [9]. The brightness of teeth and sockets of teeth in the mandible are almost the same to the naked eye, and the gradient of the teeth boundary is also unclear. Therefore, automatic teeth segmentation is very difficult. The brightness of the teeth and the sockets of teeth seem to be repeating when looking at the histogram, because they are very difficult to distinguish with the naked eye.  Figure 2 shows the histogram of CT scans of the mandible. It shows the dark background, tissues, and bones in the order from the darkest to the lightest. Bones are composed of teeth and sockets of teeth, and it is impossible to distinguish the teeth from the jawbones with a simple threshold method [10], because there are no threshold values distinguishing them.

Several approaches have been proposed for automatically separating a tooth. Chen and Jain [11] presented a method of tooth contour extraction with active contour model (ACM) [12], which uses the initial energy function and repetitively updates the contours. ACM uses the iterative method until the contours converge, so they are relatively slow and are difficult for designing the functions applying the region's geometric information. Momeni and Zoroofi [13] proposed a multistep approach for the automatic classification of teeth by using panoramic resampling/projection and level set techniques. In this method, the 2D panoramic image is generated from 3D CT volume. Then, the vertical lines separating individual teeth and the horizontal line separating the upper and lower teeth are estimated in this panoramic image. However, some teeth are not vertically aligned (e.g., molars) and cannot be accurately separated by using the rectangular region defined by those horizontal and vertical lines. Gao and Chae [14] introduced another teeth separation method by finding a plane separating two adjacent teeth in 3D space along the jaw arch. This method searches for an optimal position and orientation of the teeth separation plane, which has the minimum average intensity value. However, as it does not properly handle the soft pulp inside of the tooth, which has low intensity values, a plane crossing the soft pulp can be inappropriately detected. There have also been a couple of semiautomatic methods to detect the tooth or its axis. Gao and Chae [15] suggested a semiautomatic teeth segmentation technique using an enhanced level set method [16]. This method uses shape and intensity from the prior information of a tooth, which determines the shrinking or expanding force of the level set function. Based on the shape and intensity from the prior information on the tooth crown and root, different segmentation techniques are applied to the crown and the root. Although this technique segments a tooth with a small error, it requires users to manually draw an initial contour around each tooth which takes about as much as 5 min in addition to the time of initialization. Galanis et al. [17] proposed an approach of implant axis detection. This method finds the least square regression line fitting the centroids of the bone and prosthesis in each slice, and the uniform geometric and density distributions of the bone and prosthesis surrounding the implant axis are required. This approach requires users to specify a rectangular region around each tooth to segment the tooth region. Furthermore, the segmented tooth region includes the cortical jawbone as well as the tooth, so the tooth axis cannot be accurately detected. Moreover, there have been several attempts to solve these problems using Principal Component Analysis (PCA) [18] and Support Vector Machine (SVM) [19], which point out the features and are trained for the image segmentation. These methods not only require additional data, but also are based on the level set method, so their processing speed tends to be slow compared to the threshold method and the seeded region growing (SRG) method [20].

As earlier described, it is very difficult to segment the teeth and sockets in the mandible because their brightness is very similar and the gradient of the teeth boundary is indistinct. Most of the previous methods used only the intensity values and have no estimation for the threshold of the histogram of CT images in order to distinguish tissues and bones. Therefore, automatic teeth segmentation using earlier approach is very difficult.

In this paper, a robust algorithm is proposed that can accurately differentiate individual teeth from sockets of teeth and segment them. This method can also boost up the segmentation speed using the multiple cores. Parameter values of thresholding and shapes of the teeth are propagated to the neighboring slice, based on the separated teeth from reference images. After the propagation of threshold values and shapes of the teeth, the histogram of the current slice was analyzed. The individual teeth are automatically separated and segmented by using seeded region growing. Then, the newly generated separation information is iteratively propagated to the neighboring slice. The average error of volume measurement was 2.29 ± 0.56%, which was more accurate than the conventional methods, threshold (15.52 ± 3.08%) and region growing (7.86 ± 1.70%).

## 2. Methods

**Figure 3:**
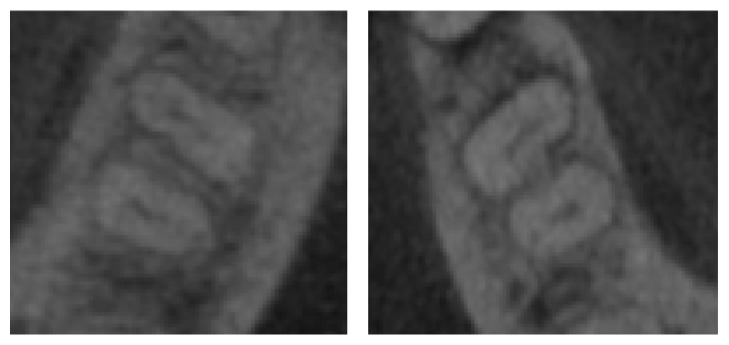
The examples of teeth images. The difference in the brightness between teeth and sockets of teeth in dental CT images is relatively low.

The following characteristic of CT scans was used in this research to differentiate teeth from sockets of teeth. First, the difference in the level of brightness between teeth and sockets of teeth is relatively low, as shown in Figure 3 [21]. Second, we initially used the threshold value [9] for distinguishing between teeth and sockets of teeth in each slice from mandibular bone. Third, we search *T*-values which easily classify teeth and sockets for proper segmentation of teeth. Finally, the shapes of teeth gradually change between slices, so it is possible to immediately stop the region extending to the sockets of teeth, when using the SRG method.

**Figure 4:**
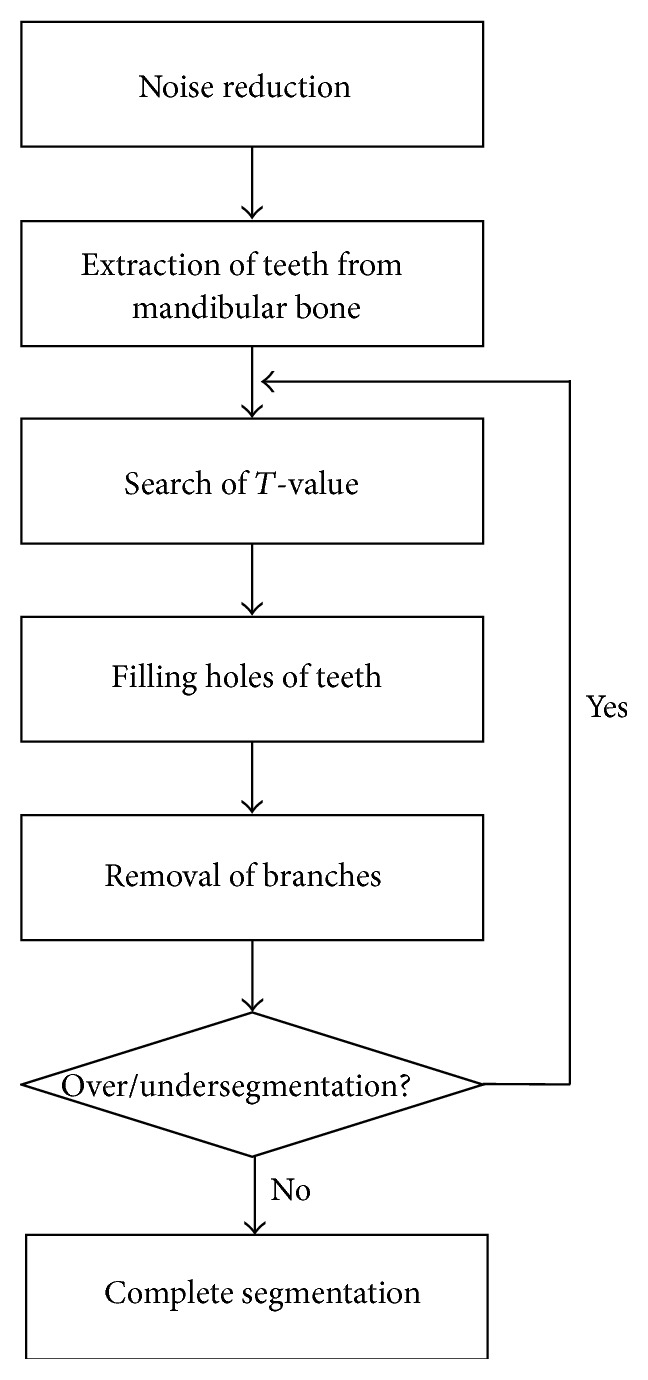
An overview of the proposed segmentation of individual teeth. First, we reduce noise and set *T*-value. Second, we fill holes of teeth and remove the branches. Finally, we check that the result of segmentation is oversegmented or undersegmented. If the result is over or under, go back to *T*-value step.

Figure 4 shows the segmentation of teeth in a slice in a flow chart. First of all, we reduced noise from the entry images by using the median filter. After that, we set the *T*-value (transmitted value from an earlier slice) as the initial value for faster search and for more accurate threshold values. In order to segment teeth more exactly, we find proper threshold by measuring the region of teeth and apply the threshold to each slice differently from the previous method [9] which applies one to the whole volume. We extended the regions using the SRG with these threshold values determined. We removed from the results the previously undiscovered branch-shaped region of the SRG. Finally, after comparing the previous slice and the size of region, we decided whether to repeat the algorithm by determining if oversegmentation and undersegmentation existed.

### 2.1. Extraction of Teeth from Mandibular Bone

**Figure 5:**
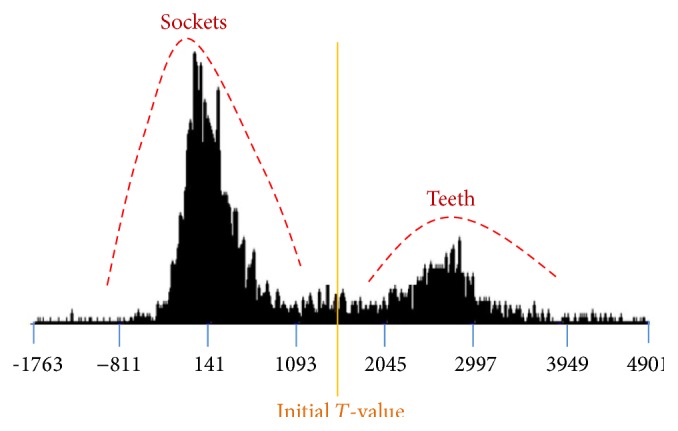
Initial *T*-value. This should be easily classified as the low density of tissues and the high density of bones.

To roughly extract teeth from mandibular bone, we used the thresholding method [22], which is the simplest method for image segmentation. During this process, each pixel (or voxel) in an image (or volume) is set as the region of interest if its value was greater than the predefined threshold value, and if otherwise, it was set as the background. Heo [21, 23] found that the threshold values for distinction between teeth and sockets of teeth slightly changed based on the slices, so he tried to find the ideal value of the threshold. He set the threshold values from the earlier slice as the initial threshold values and then reassessed the threshold values with the fewest errors. He found relatively accurate threshold values, but he still could not solve the problems of similar brightness of teeth and sockets of teeth with the threshold method that did not use the spatial information. To solve this problem, we found the proper threshold for segmentation, that is, the initial *T*-value. The initial *T*-value should be easily classified as the low density of tissues and the high density of bones, as shown in Figure 5.

Pseudocode 1 is a pseudocode for finding the initial *T*-value.

**Figure 6:**
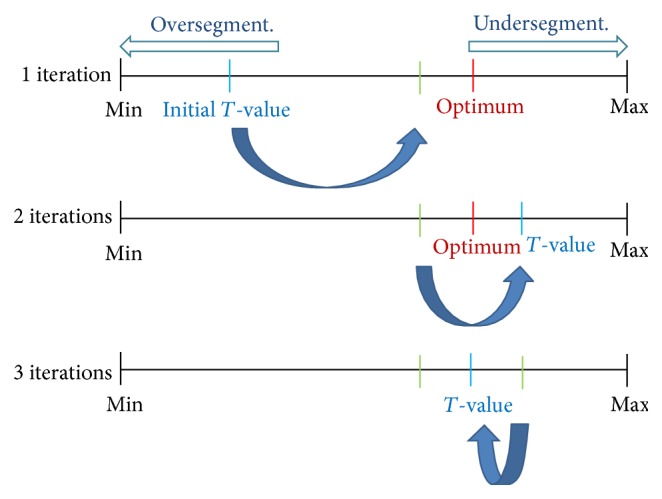
The bisection search: optimal *T*-value preventing the oversegmentation while keeping the shape and size of segmented teeth from the previous slice.

The optimum threshold value needs to be the greatest threshold value that prevents oversegmentation while keeping, to some degree, the shape and size of the segmented teeth from the previous slice. The reason for setting them as the greatest threshold value is to prevent the extension from teeth to sockets of teeth, because this would lead to an oversegmentation. We used the bisection search in order to obtain the greatest threshold value in a faster way.  Figure 6 describes the bisection search.

### 2.2. Filling Holes of Teeth

In this step, we filled the holes of teeth by using SRG. The SRG algorithm was suggested by Adams; it is relatively solid and faster and does not require particular parameters. So, it is the most used image segmentation algorithm. If the users set a part of the initial segmentation region with seed points, this algorithm outputs the connecting region that has a similar brightness to the starting point of the initial segmentation region. Akhoondali et al. [24] separated the upper jawbones from the lower jawbones by using geometric information and then set the enamel with greater brightness than the others in mandible as the threshold method. They decided to distinguish teeth from sockets of teeth using the SRG algorithm, starting from the enamel region as initial seeds. However, the brightness in enamel of each tooth was different, but it was not clearly seen in CT scans. Furthermore, if the simple SRG algorithm was applied in the normal CT data, it was usually spread to the sockets of teeth. So, they were not well segmented. Therefore, we only used SRG for filling the hole of teeth which is already roughly extracted from the mandibular bone. The algorithm's pseudocodes are as follows.

 Let *A* be a segmented region.
 Set seeds as a region *A* (seeds ∈*A*).
 
*δ* is a measure of how *x* is different from the segmented region
  (1) ∀x∈x ∣ x∉Ax⟶A,Ix−meanA<δAC,otherwise.

**Figure 7:**
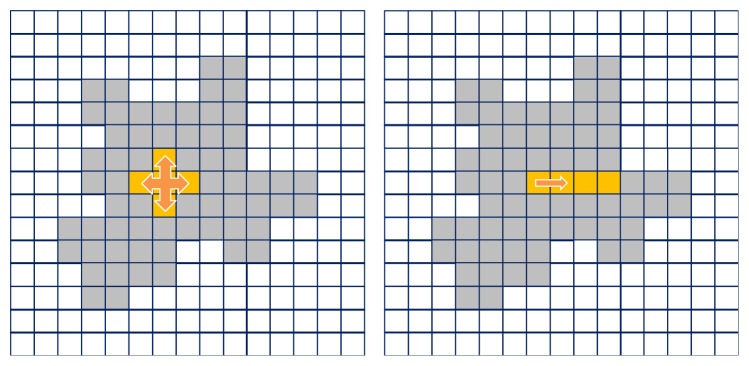
A memory access order for improving the cache efficiency. If we explored the right side, we can improve the efficiency by 5 to 10 times.

In practice, we repetitively followed the steps using the Queue data structure. In order to utilize the cache memory, we needed to grow the region while exploring *x*-, *y*-, and *z*-axis. If we explored as it was shown in the right side of Figure 7, rather than the left side, we can improve the efficiency by 5 to 10 times.

**Figure 8:**
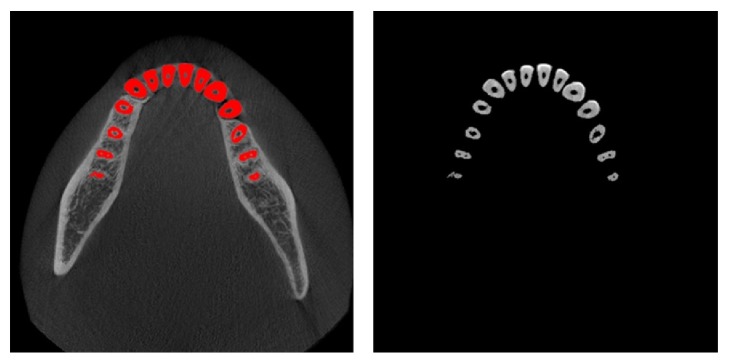
Unexpected holes shown by dentin and pulp while using simple SRG.

The brightness inside of the teeth is darker compared to the outside, and this is because of the dentin and pulp. If we simply used the SRG algorithm, the image segmentation may show teeth with holes as shown in Figure 8.

**Figure 9:**
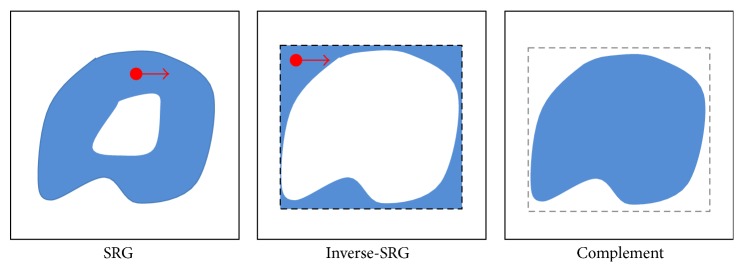
Filling the holes by SRG. We set the bounding box and fill up holes using inverse-SRG and complement operator.

**Figure 10:**
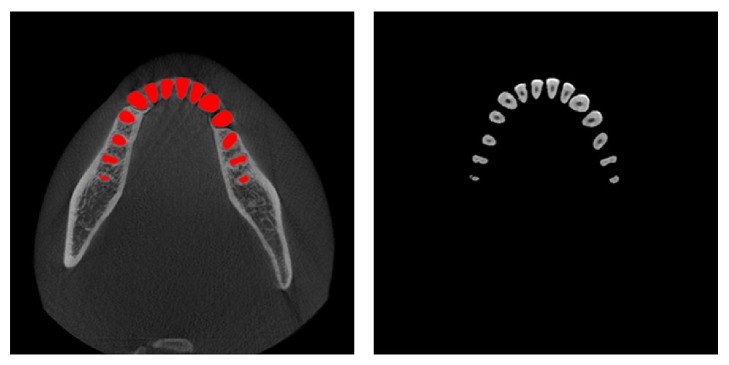
The result of teeth segmentation after filling up empty spaces.

As it is shown in Figure 9, if the region is being extended, we need to check the greatest extended region with the bounding box to fill up these holes. The boundary box is the rectangular range of the object and is defined as the extended region of each tooth. When the region extension is over, the reverse region extension will start inside of the bounding box. By reversing the region, the empty spaces will be filled up.  Figure 10 shows the image of the teeth segmentation, after filling up the empty spaces.

### 2.3. Removal of Branches in the Teeth

The segmented region in the previous slice is used for the seed points of the region growing for the next slice and also as an index to evaluate the appropriation of the segmentation. The removal of branches in the teeth is implemented as in Pseudocode 2.

**Figure 11:**
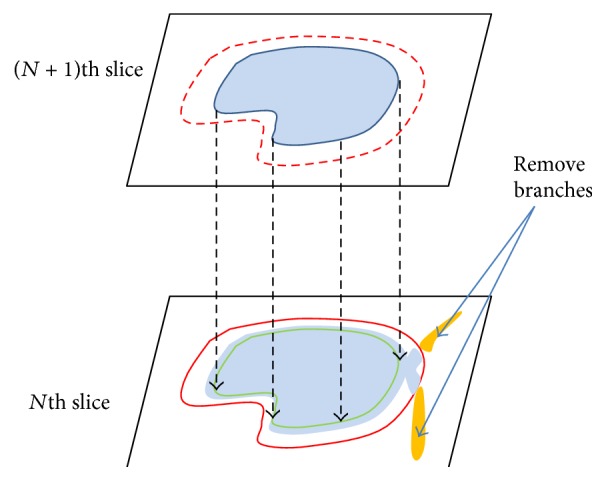
Resetting the oversegmented regions compared to the segmented region of the previous slice.

The biggest problem of the individual teeth segmentation is that the extension often spreads to the sockets of teeth or jawbones from teeth and outputs the oversegmentation. To prevent this, we need to eliminate the oversegmented regions after comparing them to the segmented region of the previous slice. For this, the dilation is operated in the previous slice by 2~5 pixels, and the oversegmented region is removed after comparing it to the current slice; this is shown in Figure 11.

### 2.4. Acceleration Using Multiple Cores

Currently, as the number of cores for CPU has been developing from 2 to 8, parallel processing techniques (multiple threads, multiple processing) are being commonly used to utilize 100% of the computing resources. To use the parallel processing, we need to create, manage, and remove each thread, but this inconvenience can be resolved by using the OpenMP library. The OpenMP API (Open Multiprocessing Application Programming Interface) supports multiple-platform shared-memory parallel programming in C/C++ and Fortran on all architectures [25].

**Figure 12:**
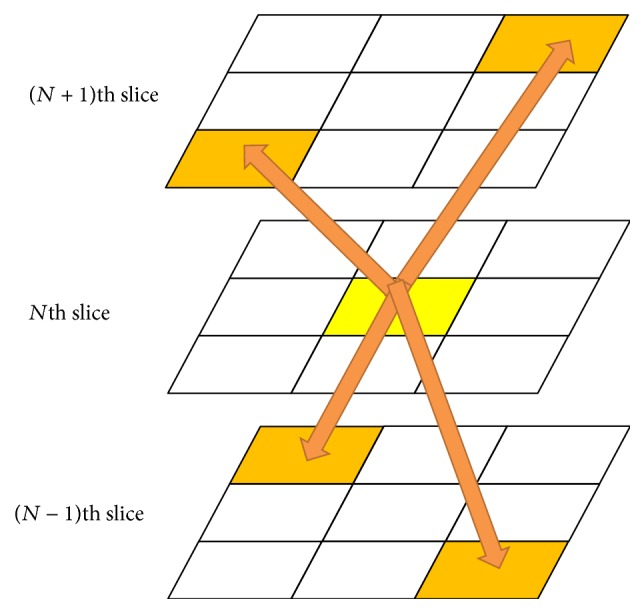
To minimize memory access conflicts among threads in the region growing algorithm, the initial seed points are spread in a 3D way.

In this research, to minimize the conflicts of memory access among threads in the region growing algorithm, we spread the initial seed points in a 3D way as shown in Figure 12.

It was possible to considerably prevent the memory access conflicts, since the region extension occurs in *x*-, *y*-, and *z*-axis directions.

## 3. Experimental Results

All evaluations were performed on an Intel Core2 Q6600 (2.4 GHz CPU) and 4 GB of main memory. Our algorithm was implemented by C++ running on Windows 7. OpenMP 3.0. API was used for parallel processing and acceleration. The proposed method has been applied to the dental CT images (512 × 512 × 400, 16-bit data). The parameters of the dental CBCT device were set at 85 kV, 4 mA, and the voxel size was 0.2 × 0.2 × 0.2 mm. The CT images were reconstructed by FDK algorithm [26].

For separating the individual tooth, the user first sets the bounding box including the area to be segmented, to determine the initial *T*-value by analyzing the histogram. Then, the user selects the seed points in the reference slice, which is clearly distinguished between teeth and sockets of teeth. Once this is completed, the proposed algorithm separates the individual teeth automatically.

### 3.1. Performance Evaluation

To validate the results of our method, we requested a dentist with ten years of clinical experience to manually segment teeth in each dataset. The dentist manually drew the boundary of teeth in axial image slices, and this was used to assess the accuracy of the proposed method. In this experiment, we used CT scans of ten people who each had 14 individual teeth in mandible.

**Figure 13:**
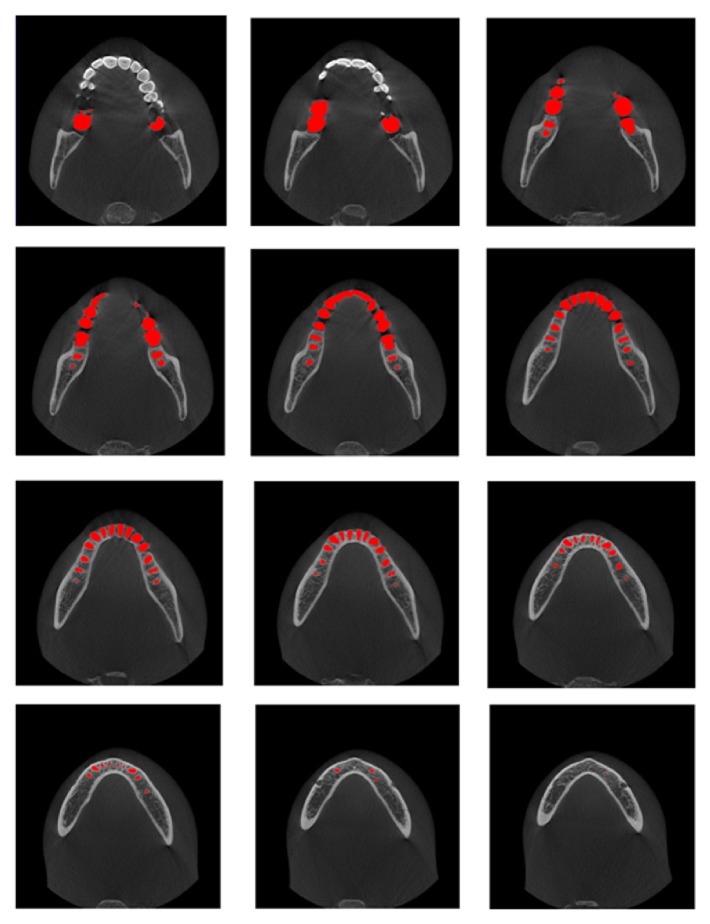
Results of segmented individual teeth by proposed method: isolated individual teeth are marked in red.

Figure 13 shows the automatically segmented mandibular teeth superimposed on the axial CT images obtained from a dataset. The isolated individual teeth were marked in red. The segmentation results were very accurate, as shown in the axial view.

Among the sequential processing steps of the proposed method, we evaluated the accuracy of the proposed method based on the four evaluation metrics as follows:

(2) Efp=numVauto−numVauto∩VmanualnumVmanual×100%,Efn=numVmanual−numVauto∩VmanualnumVmanual×100%,Evol=numVautonumVmanual−1×100%,Esim=1−2numVauto∩VmanualnumVauto+numVmanual×100%,

where *V*_auto_ and *V*_manual_ are the set of voxels in the automatically and manually segmented objects, respectively. The false positive error, *E*_fp_, is the ratio of the set of voxels in the automatically segmented object, but not in the manually segmented object, to the set of voxels in the manually segmented object. The false negative error, *E*_fn_, is the ratio of the set of voxels in the manually segmented object, but not in the automatically segmented object, to the set of voxels in the manually segmented object. The absolute volume measurement error, *E*_vol_, is the ratio between the automatically and manually segmented volumes. The similarity error, *E*_sim_, is defined by the similarity index [27].

Table 1 summarizes the segmentation errors of our teeth segmentation for ten datasets. Each value is the rate of mean ± standard deviation of all datasets. The average value of *E*_vol_ was 2.29% ± 0.56% for all datasets, indicating that the average differences between the manual and automatic teeth segmentation results were less than 3%. In addition, the average value of *E*_sim_ was 2.02% ± 0.67% for all datasets, indicating also that the average differences between the manual and automatic teeth segmentation results were less than 3%.

The average processing time of the experiments repeated three times was 3.47 sec using quad-core CPU and 8.33 sec using single-core CPU. Boosting up the speed with the quad cores was shown to be 2.4 times faster than a single core.

### 3.2. Comparison with the Previous Method

**Figure 14:**
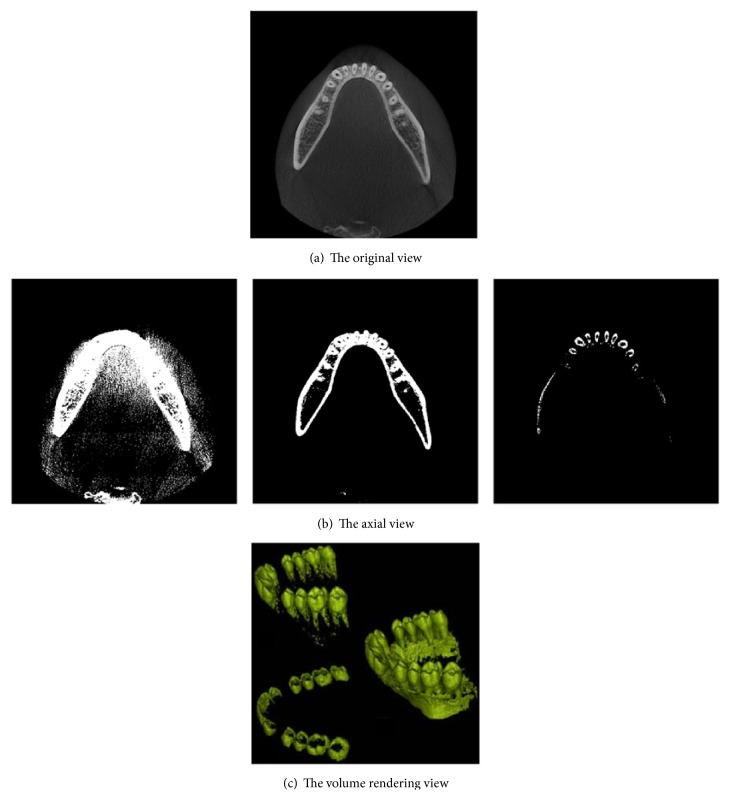
Result of threshold techniques after altering threshold values: (a) is the axial view of the original; (b) and (c) are the results of threshold techniques altering threshold values, which cause under- or oversegmentation.

Figure 14 shows the result of threshold techniques, after altering the threshold values. It is difficult to distinguish teeth from sockets of teeth by using these techniques, because they would result in either over- or undersegmentation [6].

We compared the proposed method with two state-of-the-art methods: the threshold method [6] and seed region growing [20, 24].  Table 2 shows the comparison of segmentation accuracy in these methods by using *E*_vol_. In contrast to conventional methods showing the large values of *E*_vol_, the proposed method exhibited much smaller value of *E*_vol_, indicating that the proposed method enables much more accurate teeth segmentation than conventional methods.

## 4. Conclusion and Future Work

In this paper, we suggested the way to effectively distinguish individual teeth from sockets of teeth in CT images. Threshold values and shapes of the teeth are propagated to the next slide, based on the separated teeth from reference images. After the propagated information (threshold values and shapes of teeth) and the histogram of the current slice are examined, individual teeth were automatically separated with the SRG. The individual teeth are constantly separated, as the separate information is propagated to the next slide. We physically increased the division speed by using multiple cores. Boosting up the speed with the quad cores was shown to be 2.4 times faster than a single core. Reducing segmentation time from around 9 seconds to around 4 seconds facilitates more efficient and faster clinical treatment for the dentist. The reason why the reduced computation time using quad-core CPU compared to using single-core CPU is 2.4-fold instead of 4-fold is that all the steps of our algorithm cannot be parallelized; for example, the results of previous slice are necessary in the step of removing branches.

It is difficult to extract the teeth region, especially individual teeth, because the brightness of teeth and sockets of teeth in the mandible are similar and the gradient of the teeth boundary is also unclear. In order to solve this difficulty, we segment individual teeth slice by slice using diffusion of intensity and shape of each slice. No one has researched this propagation approach to the best of our knowledge. Furthermore, computation time of our method is boosted up using the multiple cores.

A dental implant is surgically inserted into the jawbone at the location of a missing tooth. Dentists usually examine the axes of teeth and the axis of the biting tooth of the missing one in the preoperative planning of the implant placement [18]. In addition, in the dental implantology, which is in the preparation of safer and less invasive dental surgery using virtual surgery planning system [28–33], the teeth axes should be considered. To calculate the teeth axes, it is necessary to extract the jawbone and segment individual teeth from the jawbone. The region of each tooth is prerequisite to compute the teeth axes.

One limitation of the current method is that we must receive initial starting seed points and bounding box from the user. To minimize these inconveniences, we attempted to find a fully automatic segmentation method. Metal artifacts still remained as a problem in CT scans. The proposed algorithm cannot split the tooth on the metal artifacts data. In the future, we will make improvements to find the algorithm that is more robust to noise and metal artifacts.

## Figures and Tables

**Pseudocode 1:**
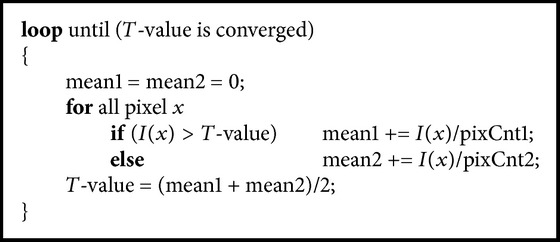


**Pseudocode 2:**
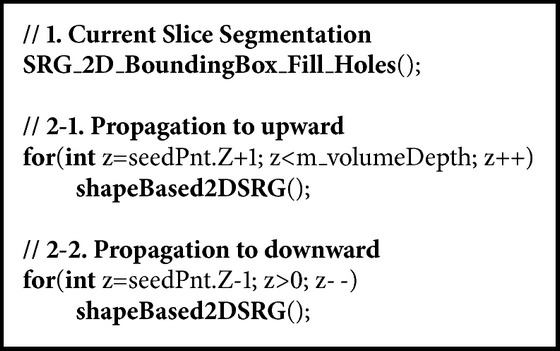


**Table 1:** Results of accuracy assessment for the proposed method.

Dataset	*E*_fp_	*E*_fn_	*E*_vol_	*E*_sim_
	(%)	(%)	(%)	(%)
1	1.36 ± 0.64	1.46 ± 1.03	1.65 ± 0.81	2.14 ± 1.90
2	2.40 ± 0.93	2.82 ± 0.84	3.19 ± 0.37	1.23 ± 0.49
3	1.43 ± 0.48	1.94 ± 0.33	2.84 ± 0.71	2.71 ± 1.05
4	2.13 ± 0.67	2.44 ± 0.77	1.72 ± 0.41	3.19 ± 0.48
5	2.80 ± 0.89	3.08 ± 1.59	2.19 ± 1.79	1.28 ± 0.52
6	2.82 ± 0.51	2.72 ± 0.92	2.09 ± 1.04	1.66 ± 1.16
7	1.65 ± 0.96	2.71 ± 1.81	2.39 ± 1.84	2.58 ± 1.25
8	2.08 ± 1.39	3.20 ± 1.82	2.06 ± 0.91	1.64 ± 0.51
9	2.87 ± 0.45	2.14 ± 0.44	1.72 ± 0.53	1.44 ± 0.41
10	2.51 ± 1.18	2.11 ± 0.89	3.03 ± 1.07	2.30 ± 1.55

**Table 2:** Comparison of segmentation accuracy, *E*_vol_.

Dataset	Threshold	Region growing	Proposed method
1	10.39 ± 5.13	6.09 ± 3.36	1.65 ± 0.81
2	16.57 ± 3.96	6.60 ± 5.27	3.19 ± 0.37
3	18.12 ± 5.99	9.87 ± 3.03	2.84 ± 0.71
4	15.38 ± 7.13	9.75 ± 3.52	1.72 ± 0.41
5	10.27 ± 6.27	6.12 ± 5.15	2.19 ± 1.79
6	19.43 ± 3.18	8.71 ± 5.95	2.09 ± 1.04
7	16.43 ± 7.27	6.61 ± 4.76	2.39 ± 1.84
8	14.37 ± 7.20	9.79 ± 3.29	2.06 ± 0.91
9	17.88 ± 6.53	6.04 ± 5.37	1.72 ± 0.53
10	16.37 ± 6.03	8.98 ± 3.74	3.03 ± 1.07

## Appendix

### Pseudocodes of Proposed Method

Input is the input CT images.

Output is the final segmentation map.

(i) Denoise the input images with Gaussian and median filtering.
(ii) Extract the teeth from mandibular bone with thresholding method.
(iii) While the segmentation result is over- or undersegmentation,
  (a) search *T*-value for distinction between teeth and sockets,
  (b) fill holes of teeth using SRG algorithm,
  (c) remove the branches in the teeth using comparison with the segmented region of the previous slice.
(iv) Complete segmentation of the teeth.
